# Multiobjective Memetic Estimation of Distribution Algorithm Based on an Incremental Tournament Local Searcher

**DOI:** 10.1155/2014/836272

**Published:** 2014-07-23

**Authors:** Kaifeng Yang, Li Mu, Dongdong Yang, Feng Zou, Lei Wang, Qiaoyong Jiang

**Affiliations:** ^1^School of Computer Science and Engineering, Xi'an University of Technology, P.O. Box 666, No. 5 South Jinhua Road, Xi'an 710048, China; ^2^School of Computer Science, Xi'an Polytechnic University, China; ^3^Shaanxi Huanghe Group Co., Ltd., Xi'an, China

## Abstract

A novel hybrid multiobjective algorithm is presented in this paper, which combines a new multiobjective estimation of distribution algorithm, an efficient local searcher and *ε*-dominance. Besides, two multiobjective problems with variable linkages strictly based on manifold distribution are proposed. The Pareto set to the continuous multiobjective optimization problems, in the decision space, is a piecewise low-dimensional continuous manifold. The regularity by the manifold features just build probability distribution model by globally statistical information from the population, yet, the efficiency of promising individuals is not well exploited, which is not beneficial to search and optimization process. Hereby, an incremental tournament local searcher is designed to exploit local information efficiently and accelerate convergence to the true Pareto-optimal front. Besides, since *ε*-dominance is a strategy that can make multiobjective algorithm gain well distributed solutions and has low computational complexity, *ε*-dominance and the incremental tournament local searcher are combined here. The novel memetic multiobjective estimation of distribution algorithm, MMEDA, was proposed accordingly. The algorithm is validated by experiment on twenty-two test problems with and without variable linkages of diverse complexities. Compared with three state-of-the-art multiobjective optimization algorithms, our algorithm achieves comparable results in terms of convergence and diversity metrics.

## 1. Introduction

Multiobjective optimization usually involves many conflicting, incomparable, and noncommensurable objectives. During the past two decades, multiobjective evolutionary algorithms (MOEAs) have obtained much more interest among optimization community mainly because of the fact that they can be suitably applied to deal simultaneously with a set of possible solutions. A number of evolutionary algorithms have been developed for multiobjective problems, such as strength Pareto evolutionary algorithm (SPEA) [1], Pareto archived evolution strategy (PAES) [2], Pareto envelope-based selection algorithm (PESA) [3, 4], micro-GA [5], nondominated sorting genetic algorithm II (NSGA-II) [6], strength Pareto evolutionary algorithm 2 (SPEA2) [7], multiple objectives with particle swarm optimization (MOPSO) [8], nondominated neighbor immune algorithm (NNIA) [9], and MOEA with adaptive weight adjustment (MOEA/D-AWA) [10]. Following the recent review of evolutionary multiobjective optimization fields [11, 12], NSGA-II and SPEA2 can be considered as two representatives of state-of-the-art MOEAs in current multiobjective optimization community.

The current MOEAs research mainly focuses on the following several highly related issues, such as fitness assignment, diversity maintenance, external population, combination of MOEA and local search, new dominance scheme, and many-objective problems. However, there are little fresh work done on how to generate new solutions and most current MOEAs directly adopt traditional crossover and mutation operators. Based on the works [13, 14], we can gain multiobjective problems (MOPs) with variable linkages causing trouble to MOEAs with traditional crossover and mutation operators. It seems that it is urgent to design new scheme to generate new solutions.

Estimation of distribution algorithms (EDAs) are a new computing paradigm in evolutionary computation. A posterior probability distribution model based on globally statistical information from the selected solutions is built to generate new solutions for next generation. This new class of algorithms generalizes genetic algorithms by replacing the crossover and mutation operators by learning and sampling the probability distribution of the promising individuals of population per iteration. Working in such a way, the relationships between the variables involved in the problem domain are explicitly and effectively captured and exploited. Several EDAs have been proposed for MOPs. Khan [15] proposed multiobjective BOA (mBOA) and multiobjective hierarchical BOA (mhBOA) by combining the model building and model sampling procedures of BOA [16] and hierarchical BOA (hBOA) [17] with the selection procedure of NSGA-II. They compared the performance of mBOA and mhBOA with that of NSGA-II on a class of bounded difficult additively separable deceptive and hierarchically deceptive functions. Bosman and Thierens [18] combined IDEAs with nondominated tournament selection and clustering, and they used clustering to split the population into subpopulation and separate models were built for each subpopulation. Laumanns and Ocenasek [19] combined mixed BOA with the selection and replacement procedures of SPEA2. The algorithm was tested on some knapsack problems, and it was shown to dominate NSGA-II and SPEA2 in most instances. Zhou et al. [20] present a way, named multiobjective EDA based on decomposition, which utilizes decomposition based techniques and probabilistic model based methods, to tackle the traveling salesman problem. However, these MOEDAs do not involve how the Pareto set distributes in the decision space.

The Pareto set of a continuous MOP is a piecewise continuous (*m* − 1)-dimensional manifold, where *m* is the number of the objectives, which have been investigated and applied by several scholars [21, 22]. Zhang et al. [14] exploited the property explicitly and proposed regularity model based multiobjective estimation of distribution algorithm, called RM-MEDA, a regularity model based EDA for continuous MOPs. They have validated that RM-MEDA can effectively deal with MOPs with variable linkages and admitted that the performance of RM-MEDA declines if a MOP has many local Pareto fronts [14, 23].

As Ong et al. [24, 25] suggested, in recent years, the development of hybrid genetic algorithm is one of the most significant trends in the field of metaheuristics. Methods of this kind hybridize recombination operators with local heuristics. Such a hybrid algorithm is also called a memetic algorithm. It is clearly shown that memetic algorithms have higher search ability than traditional EMO algorithms [26]. Ishibuchi and Murata [27] are the first two scholars to propose a multiobjective genetic local search algorithm. Afterward, Xu et al. [28] proposed GLSA, Jaszkiewicz [29] proposed MOGLS, and Liu et al. [30] proposed FMOPSO. As forenamed, RM-MEDA extracts globally statistical information to build the probability distribution model and performs weakly in local search. If we hybridize an effective local search operator with RM-MEDA, we may get balance between exploration and exploitation in the search space. For this end, an incremental tournament local searcher operator is proposed in our study, which biases solutions with high isolation value. Our algorithm is called multiobjective memetic estimation of distribution algorithm.

In order to keep well-spread Pareto-optimal solutions, *ε*-dominance is employed in our algorithm. The *ε*-dominance does not allow two solutions with a difference less than *ε*_*i*_ in the *i*th objective to be nondominated to each other, thereby allowing a good diversity to be maintained in a population. Besides, the method is quite pragmatic because it allows the user to choose a suitable *ε*_*i*_ depending on the desired resolution in the *i*th objective. Deb et al. [31] have validated that the diversity metric of *ε*-MOEA is slightly better than that of NSGA-II based on crowding distance.

Moreover, in [14], Zhang et al. proposed ten multiobjective problems with variable linkages. However, distribution of these ten problems is linear or almost linear in decision space. Besides, RM-MEDA introduces local principal component analysis to build probability distribution model, which is linear or local-linear distribution in decision space. Therefore, most of these problems may be easy for RM-MEDA. For this end, two more difficulty problems are proposed in this paper, which more accord with manifold distribution in the decision space by introducing nonlinear mapping into variables.

In the remainder of the paper, we briefly mention notations and definitions in Section 2. Thereafter, in Section 3, we briefly describe RM-MEDA.  Section 4 presents the proposed MMEDA.  Section 5 describes our proposed two problems. In Section 6, the experimental study is demonstrated. Finally, we outline the conclusions of this paper.

## 2. Definitions and Notations

In our paper, we consider the following continuous MOP:

(1) $$\begin{array}{c} \min f\left(x\right)={\left(f_{1}\left(x\right),f_{2}\left(x\right),\ldots ,f_{m}\left(x\right)\right)}^{T}, \end{array}$$

where **x** ∈ Ω⊆*R* ^*N*^, **x** is a decision variable vector, and Ω is a continuous search space. *f* : **x** → *R* ^*m*^ is the map of decision variable space to *m* real valued objective space. The objectives in a MOP conflict each other, and no single solution can optimize all the objectives at the same time. The Pareto front/Pareto set (PF/PS) is set of all the optimal tradeoff solutions in the objective/decision space.

**Definition 1 (Pareto dominance).**

There is a vector **u** = (*u*_1_, *u*_2_,…, *u*_*m*_), which is said to dominate **v** = (*v*_1_, *v*_2_,…, *v*_*m*_) (denoted by **u** < **v**), if and only if **u** is partially less than **v**, which is equal to this expression: ∀*i* ∈ {1,…, *m*}, *u*_*i*_ ≤ *v*_*i*_∧∃*i* ∈ {1,…, *m*} : *u*_*i*_ < *v*_*i*_.

**Definition 2 (Pareto optimality).**

A point x* ∈ Ω is a random optimal point and if for every **x** ∈ Ω and **I** = (1,2,…, *m*) either ∀*i* ∈ **I**, *f*_*i*_(**x**) = *f*_i_*(**x**) or there is at least one *i* ∈ **I** such that *f*_*i*_(**x**) > *f*_i_*(**x**). In other words, this definition says that x* is Pareto optimal if there are no feasible vectors **x** which can decrease some criterion without causing a simultaneous increase in at least one other criterion.

## 3. The Framework of RM-MEDA

**Figure 1:**
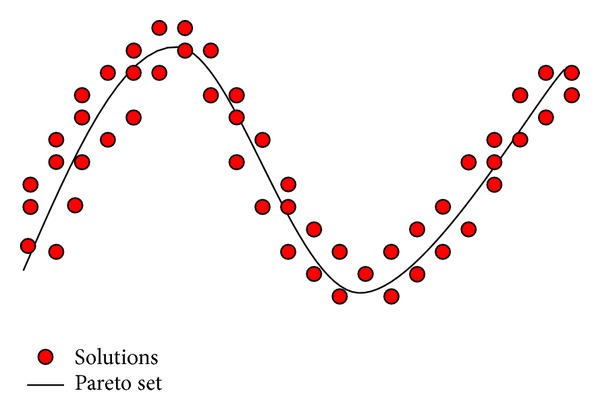
Illustration of individual solutions scattered around PS in the decision space.

Under some smoothness assumptions, it could be induced from the Karush-Kuhn-Tucker condition that the PS of (1) defines a piecewise continuous (*m* − 1)-dimensional manifold, where *m* is the number of objectives. In [14], they give us the following model to illustrate individual solutions scattered around the PS in the decision space:

(2) $$\begin{array}{c} \xi =\zeta +\nu , \end{array}$$

where *ζ* is uniformly distributed over a piecewise continuous (*m* − 1)-dimensional manifold. *ν* is an *n*-dimensional zero-mean noise vector and *n* is the number of decision variables.  Figure 1 illustrates the basic idea of RM-MEDA.

In [14], piecewise (*m* − 1)-dimensional linear models are used to approximate model *ζ* in (2). Local principal component analysis is applied to partition population. In RM-MEDA, the number of clusters is an experimental parameter and Zhang sets it to be 5. The solutions of each cluster are used to build a statistical model by principal component analysis and we could get the parameters of each model and noise *ν* in (2). New trial solutions are sampled from each local model.

As mentioned above, offspring solutions are generated by the statistical model and how to build the model is crucial to this algorithm. In RM-MEDA, they first partition population *P*(*t*) into disjoint clusters *S* ^1^, *S* ^2^,…, *S* ^*K*^ by local principal component analysis, and more details about partition can be found in [32]. For each cluster *S* ^*j*^, let ${\overset{-}{x}}^{j}$ be its mean and **U**_*i*_^*j*^ its *i*th principal component. Compute following two equations:

(3) $$\begin{array}{c} a_{i}^{j}=\min{\left(x-{\overset{-}{x}}^{j}\right)}^{T}U_{i}^{j},\\ b_{i}^{j}=\max{\left(x-{\overset{-}{x}}^{j}\right)}^{T}U_{i}^{j},\;x\in S^{j} \end{array}$$

for *i* = 1, 2, …, *m*. Then, set

(4) Ψj={x∈Rnx=x−j+∑αiUij, aij−0.25(bij−aij) ≤αi≤bij+0.25(bij−aij), i=1,2,…,m−1}.

Let *λ*_*i*_^*j*^ be the *i*th largest eigenvalue of covariance matrix of points in *S* ^*j*^, and *ν*~*N*(0, *δ*_*j*_ **I**):

(5) $$\begin{array}{c} \delta_{j}=\frac{1}{n-m+1}\left(\lambda_{m}^{j}+\lambda_{m+1}^{j}+\cdots +\lambda_{n}^{j}\right). \end{array}$$

From (4) and (5), we can get the model of (2) for each cluster, and new offspring solutions are sampled from the model. The flowchart of RM-MEDA is illustrated in Algorithm 1.

RM-MEDA is a novel and efficient multiobjective estimation of distribution algorithms and has been validated by multiobjective problems with variable linkages. However, it may fail in locating the global PF if a MOP has many local PFs [23], and it has not been tested by famous ZDT and DTLZ problems. In the next section, we proposed multiobjective memetic estimation of distribution algorithms, which is a more efficient and effective hybrid multiobjective algorithm.

## 4. The Proposed Method: MMEDA

### 4.1. Incremental Tournament Local Searcher (ITLS)

It is known that memetic algorithms, combined with EAs and local search heuristics, can be implemented to maintain a balance between exploration and exploitation in the search space. Besides, it is clearly shown by Jaszkiewicz [26] that memetic EMO algorithms have higher search ability than traditional EMO algorithms. Several memetic EMO algorithms have been proposed and show high competitive performance. In this paper, an incremental tournament local searcher is proposed and combined with RM-MEDA.

Following Liu et al.'s recent reviews [30], the issues considered in the design of the local searcher operator include (1) the selection of appropriate search direction; (2) the selection of appropriate solutions for local optimization; and (3) the allocation of computational budget for local search. With these notions in mind, we devise our local searcher as follows.

**Figure 2:**
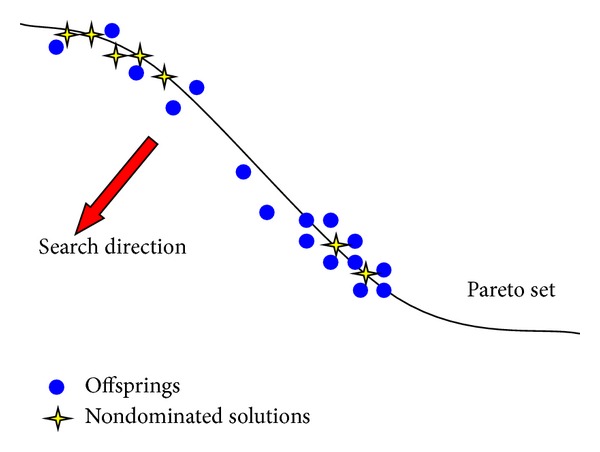
Illustration of basic idea of ITLS.

An active subpopulation is selected by tournament; that is, *N*_*s*_ nondominated solutions are selected before modeling in RM-MEDA per iteration. At first, a tournament pool is built by nondominated solutions with higher value of crowding distance, and the size of tournament pool is *n*_*s*_. If the number of nondominated solutions found so far is less than *n*_*s*_, all of the nondominated solutions are included in the tournament pool. Then we select solutions with higher value of crowding distance by performing tournament selection, and the winner solutions are put in the active subpopulation, whose size is *N*_*s*_. Since *N*_*s*_ is always larger than the size of tournament pool, *n*_*s*_, we call it incremental tournament local searcher. Then, traditional genetic operators are employed on the active subpopulation for local search.  Figure 2 illustrates the basic idea of ITLS.

By Figure 2, we can obtain that if a nondominated solution locates in more isolated regions, there are more offsprings created near the solutions. The aim is that the larger the crowding-distance value of an individual, the more the times the individual will be reproduced. So there exist more chances to do search in less-crowded regions of the tradeoff fronts. Now, we can answer the former three issues of local searcher. The search direction is optimization direction and is illustrated in Figure 2; the solutions for local optimization are biased towards isolated ones; and the allocation of computational resource for local searcher is determined by *N*_*s*_, the size of active subpopulation.

In order to present ITLS in detail, the procedure is outlined in Pseudocode 1. Before we describe the ITLS, let us give some more notations. The tournament scale is *n*_*t*_, and population *P*(*t*) is from RM-MEDA before modeling. Nondominated solutions from *P*(*t*) are denoted by *N*(*t*).

**Pseudocode 1:**
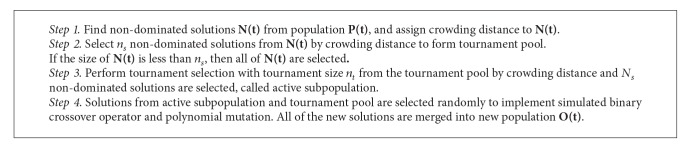
The main pseudocode of ITLS.

### 4.2. The Adopted *ε*-Dominance

It is clear from the existing studies that there are two distinct goals in the development of an MOEA: the first one is convergence to the true Pareto-optimal front; then the second one is maintenance of a well-distributed set of nondominated solutions. The proposed ITLS in former subsection corresponds to the first goal. In order to maintain well-spread Pareto-optimal solutions, we introduce the *ε*-dominance in our paper.

The *ε*-dominance does not allow two solutions with a difference less than *ε*_*i*_ in the *i*th objective to be nondominated to each other, thereby allowing a good diversity to be maintained in a population. By *ε*-dominance, the search space is divided into a number of grids (or hyperboxes) and the diversity is maintained by ensuring that a grid or hyperbox can be occupied by only one solution. In our MMEDA, there are two coevolving populations: an internal population*P*(*t*) and an archive population *N*(*t*). The archive population is updated based on the *ε*-dominance concept, whereas a usual domination concept is used to update the internal population. Every solution in the archive is assigned an identification array (**B** = (*B*_1_,*B*_2_,…,*B*_*m*_)^*T*^, where *m* is the total number of objectives) as follows:

(6) $$\begin{array}{c} B_{j}\left(f\right)=\left\lfloor \frac{\left(f_{j}-f_{j}^{\min}\right)}{\varepsilon_{j}}\right\rfloor \;\text{for}\;\min\;f\left(x\right)\;\text{in}\;\left(1\right), \end{array}$$

where *f*_*j*_^min⁡^ is the minimum possible value of the *j*th objective (default value of it is *f*_*j*_^min⁡^ = 0) and *ε*_*j*_ is the allowable tolerance in the *j*th objective below which two values are meaningless to the user. With the identification arrays calculated for the offspring and each archive member, we can decide how the archive population updates. More details of *ε*-dominance can be found in [31].

### 4.3. Details of MMEDA

As mentioned above, RM-MEDA extracts globally statistical information to build the probability distribution model and then samples it to generate offspring, which emphasizes global statistics ability, yet brings the trouble of weak performance in local search. However, several memetic MOEAs have been proposed and implemented to maintain a balance between exploration and exploitation in search space, which is often crucial to the success of the search and optimization process. For this end, a local search operator, called ITLS, is proposed to combine with RM-MEDA. Besides, in order to maintain well-spread Pareto-optimal solutions, *ε*-dominance is employed in our algorithm, whose name is MMEDA. The proposed algorithm is hybrid with global search and local search, and the success of it is due to the tradeoff between the exploration ability of RM-MEDA and the exploitation ability of ITLS. The details of MMEDA are in Algorithm 2.

## 5. Two Novel Multiobjective Problems with Variable Linkages

A number of test problems for multiobjective optimization have been designed by some scholars, such as SCH by Schaffer [33], KUR by Kursawe [34], FON by Fonseca and Fleming [35], ZDTs by Zitzler et al. [36], and DTLZs by Deb et al. [37]. These MOPs have been used in a number of significant past studies of EMO to test an optimization methodology. Following Deb's recent review of multiobjective test problems, many of the existing test problems are separable variable-wise or possess linear functions of the variable, which may not provide adequate difficulties to an EMO methodology. Zhang et al. [14] realized the point and proposed ten multiobjective problems with variable linkages (F1~F10 in Table 1).

Analyzing the ten problems proposed by [14], we can get that **x**_*i*_ = **x**_1_, *i* = 2,…, 30, for F1~F4, and $x_{i}=\sqrt{x_{1}}$ , *i* = 2,…, 30, for F5~F10, when all the solutions converge to Pareto- optimal fronts. The scheme of introducing variable linkages proposed in the ten problems can be regarded as variable linear or near-linear mapping. Besides, RM-MEDA is based on probability distributions model built by local principal component analysis, which is also a linear or local-linear statistical model. Consequently, RM-MEDA may be efficient to most of these problems. In order to investigate the performance of the optimization methodology, more problems should be employed and tested. For this end, we introduce twenty-two multiobjective problems with and without variable linkages in our experiment in Table 1.

The variable linkages in our proposed problems are based on the following nonlinear mapping on the variables:

(7) $$\begin{array}{c} x_{1}⟶x_{1},\;\;x_{i}⟶\sin\left(\pi x_{i}\right)-x_{1},\;i=2,\ldots ,n. \end{array}$$

In Figure 1, we can obtain that the Pareto set, in the decision space, of a continuous multiobjective optimization problem is a piecewise continuous (*m* − 1)-dimensional manifold, and it seems that the ten problems (F1~F10) may be a simple and special case of the regularity. The problem introduced by nonlinear mapping in (7) seems more accordant with the regularity. Furthermore, there is no common rules of why we choose sin() function, and other nonlinear function can be used, and more difficulty problems can be proposed by modifying the period of the sin(). The details of two advanced problems are in Table 1 (AF1 and AF2).

## 6. Experimental Study

### 6.1. Test Problems

The first five ZDT problems were developed by Zitzler et al. [36] (so called ZDT problems), and the next five DTLZ problems were defined by Deb et al. [37] (so called DTLZ problems). These problems have been cited in a number of significant past studies in EMO community and they can test evolutionary multiobjective optimization algorithms in different aspects. Furthermore, ten problems with variable linkages proposed by Zhang et al. [14] have also been introduced in our paper, which can bring trouble to most of variable-wise EMO methodology, and have been validated by Zhang et al. Lastly, two more difficult problems proposed by ourselves are also presented in Table 1.

It is necessary to note that the performance of an MOEA in tackling multiobjective constrained optimization problems may largely depend on the constraint-handling technique used [38], so we do not mention side-constrained problems in this study.

### 6.2. Performance Metrics

Zitzler et al. [39] suggested that for a *k*-objective optimization problem, at least *k* performances are needed to compare two or more solutions and an infinite number of metrics to compare two or more sets of solutions. Coello Coello et al. [8] proposed three issues to allow a quantitative assessment of the performance of a multiobjective optimization algorithm. As we know, it is a common task for any multiobjective optimization algorithm to find solutions as close as possible to the Pareto front and to make them as diverse as possible in the obtained nondominated front. Furthermore, the latter case includes maximizing the spread and the uniformity of solutions found in the final Pareto front. For this end, three metrics are employed in our paper to investigate the performance of the algorithm. As [14], we apply inverted generation distance to the final Pareto-optimal set obtained by an MOEA to evaluate its convergence and spread performance. We also adopt convergence metric proposed by Deb et al. [6] to measure the convergence to the final solutions. Finally, in order to check the uniformity of Pareto-optimal solutions we get in final generation and spacing devised by Schott [40] metric is employed in our paper. The three metrics are summarized as follows.

#### 6.2.1. Inverted Generation Distance

Let P* be a set of uniformly distributed points in the objective space along the PF. Let *P* be an approximation to the PF; the inverted generational distance from P* to *P* is defined as

(8) $$\begin{array}{c} D\left(P^{*},P\right)=\frac{\sum_{v\in P^{*}}d\left(v,P\right)}{\left|P^{*}\right|}, \end{array}$$

where *d*(*v*, *P*) is the minimum Euclidean distance between *v* and the points in *P*. The inverted generation distance denotes the metric convergence and spread, which represents the distance between the set of the true Pareto-optimal fronts and converged Pareto solutions obtained by EMOAs.

#### 6.2.2. Convergence Metric

Metric *Y* is used to estimate how far the elements in the set of nondominated solutions found so far are from those in the Pareto-optimal set, and it is defined as

(9) $$\begin{array}{c} Y\left(P,P^{*}\right)=\frac{\sum_{v\in P}d\left(v,P^{*}\right)}{\left|P\right|}, \end{array}$$

where *d*(*v*, P*) is the minimum Euclidean distance between *v* and the points in P*. Since multiobjective algorithms can be tested on problems having a known set of Pareto-optimal solutions, the calculation of the two metrics is possible.

#### 6.2.3. Spacing

Let *P* be the final approximate Pareto-optimal set obtained by an MOEA. The function *S* is as follows:

(10) $$\begin{array}{c} S=\sqrt{\frac{1}{h-1}\overset{h}{\underset{i=1}{\sum }}{\left(\overset{-}{d}-d_{i}\right)}^{2}}, \end{array}$$

where *d*_*i*_ = min⁡_*j*_⁡(|*f*_1_^*i*^(*x*) − *f*_1_^*j*^(*x*)| + ⋯+|*f*_*m*_^*i*^(*x*) − *f*_*m*_^*j*^(*x*)|), $\overset{-}{d}$ is the mean of all *d*_*i*_, and *h* is the number of nondominated solutions found so far. A value of zero for this metric indicates that all members of the Pareto front currently available are equidistantly spaced.

### 6.3. The Compared Algorithms

As Deb et al.'s suggestion in [13], some EMO procedures with variable-wise recombination operators do not perform as well as those with vector-wise operators; besides, PCX-NSGA-II [13] and GDE3 [41] are recommended to handle linkages-based multiobjective optimization problems. Zhang et al. [14] compared RM-MEDA with GDE3, PCX-NSGA-II, and MIDEA [42]. They concluded that RM-MEDA performed better than GDE3, PCX-NSGA-II, and MIDEA on some test instances with variable linkages, and MIDEA performed slightly badly among the four algorithms. Overall considering former works [13, 14], we choose RM-MEDA, GDE3, and PCX-NSGA-II as the comparisons with MMEDA.

The third evolution version of generalized differential evolution (GDE3) is another updated version of original DE, which modifies earlier GDE version using a growing population and nondominated sorting with pruning of nondominated solutions to decrease the population size at each generation. The procedure is similar to NSGA-II except that the simulator binary crossover is replaced with a differential evolution operator. The code of GDE3 used in comparison is written in MATLAB by the authors.

Parent-centric recombination (PCX) is a real parameter genetic operator in [43]. Deb et al. [13] introduced the PCX recombination operator in NSGA-II and validated that PCX-based NSGA-II performs better on some problem with variable linkages. The details of PCX-NSGA-II can be found in [13]. The code of PCX-NSGA-II is programmed by ourselves by referring to the code of SBX-NSGA-II and G3PCX from KanGAL (http://www.iitk.ac.in//kangal/).

We have discussed so much about RM-MEDA in the former section in our paper. Here, we acknowledge the help of Professor Qingfu Zhang and Dr. Aimin Zhou for sharing the code of RM-MEDA with us and their insightful comments.

### 6.4. Experimental Setup

In our experiments, the code is programmed in MATLAB. The source code of MMEDA can be obtained from the authors upon request. All the simulations run at a personal computer with P-IV 3.0 G CPU and 2 G RAM. The experimental setting is as follows. Firstly, in GDE3, both CR and F in the differential operator are set to be 1 for F1~F10 and AF1~AF3, which have been investigated by Deb et al. [13] and Zhang et al. [14] on MOPs with variable linkages. Furthermore, by referring to the involved literatures [44, 45], CR and F are set to be 0.2 for ZDTs and DTLZ2, DTLZ4, and DTLZ6 except 0.5 for DTLZ1 and DTLZ3. Secondly, in PCX-NSGA-II, σ in PCX is set to be 0.4 for all the test instances which work well in studies in [13]. Thirdly, in RM-MEDA, all the parameters' setting is the same as the original paper; that is, in local PCA algorithm, *K* is set to be 5, and extension rate is 0.25. Finally, in MMEDA, the size of tournament pool, *n*_*s*_, is 30, tournament scale is 2, and the size of active subpopulation, *N*_*s*_, is 40 for all test instances. Besides, the crossover probability of *p*_*c*_ = 0.9 and a mutation probability of *p*_*m*_ = 1/*r* (where *r* is the number of decision variables for real-coded GAs) for test instances. We choose *η*_*c*_ = 15 and *η*_*m*_ = 20, which are similar to Deb et al.'s setting in [6].

Furthermore, the fourth column in Table 1 is the number of total evaluations for each test instance, respectively, which is followed by some significant past studies in this area [9, 14, 37, 46]. Indexes of the different algorithms are shown in Table 2. The *ε* values for different test instances are described in Table 3, which are similar to *ε*-MOEA [31] and *ε*-ODEMO [47]. Besides, we select 500 for two objective problems and 1000 for three objective problems with evenly distributed points in Pareto-optimal front, and these points are denoted by P*.

### 6.5. Experimental Results of Multiobjective Problems without Variable Linkages

Deb et al. [13] and Zhang et al. [14] have investigated and validated that GDE3, PCX-NSGA-II, and RM-MEDA performed better than other EMOAs on multiobjective problems with variable linkages. However, whether these algorithms could get the same good performance on multiobjective problems without variable linkages is still not investigated so far. For this end, we give the experimental comparison of MMEDA, RM-MEDA, GDE3, and PCX-NSGA-II on famous ZDT and DTLZ problems in this section.

**Figure 3:**
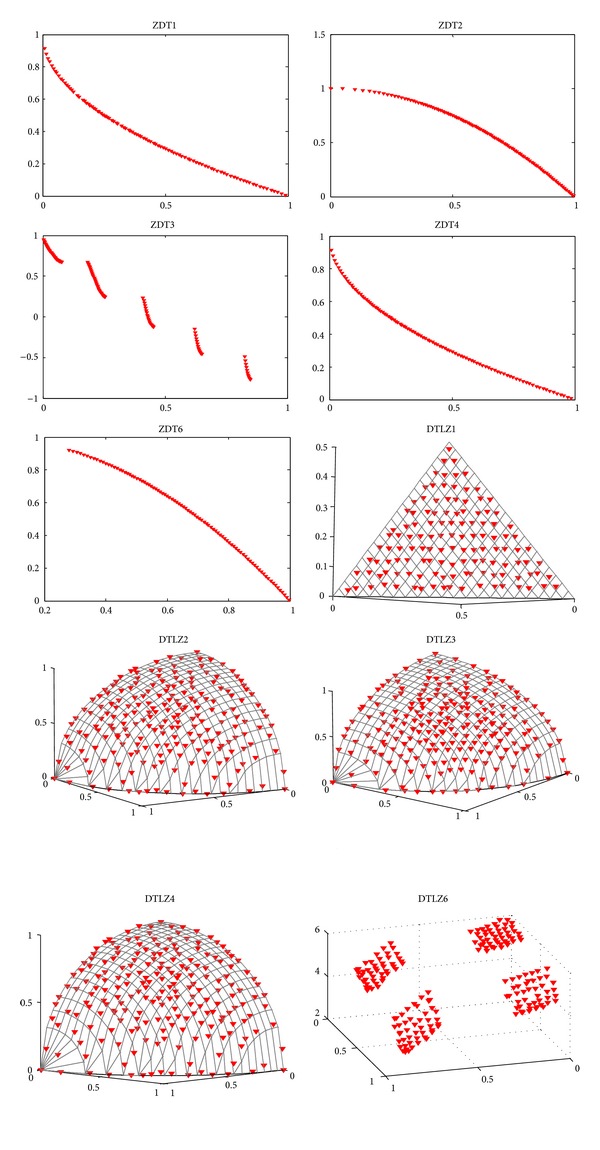
Pareto fronts obtained by MMEDA on ZDT and DTLZ problems in our paper, respectively.

Figure 3 shows the Pareto fronts obtained from our algorithm in a random single run. These problems without variable linkages are rarely tested by estimation of distribution algorithms, and we can see that our method could get fairish results on famous ZDT and DTLZ test instances. Besides, seeing DTLZ2, DTLZ3, and DTLZ4 in Figure 3, we can gain that some extreme points of the Pareto front, as well as points located in segments of the Pareto front that are almost horizontal or vertical, are lost. This is the curse of *ε*-dominance, which has been investigated by Deb et al. [31] and Hernández-Díaz et al. [48].

**Figure 4:**
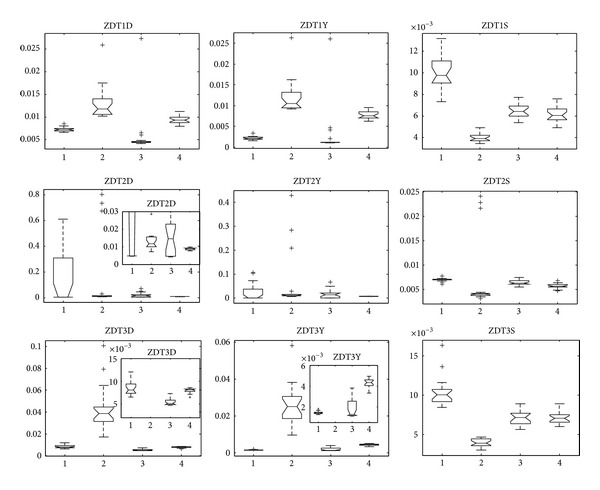
Statistical values of inverted generation distance (“D” in ZDTXD for short), convergence (“Y” in ZDTXY for short), and spacing (“S” in ZDTXS for short) for ZDT1, ZDT2, and ZDT3 obtained by MMEDA, RM-MEDA, GDE3, and PCX-NSGA-II, respectively.

Next, we investigate statistical results of the four algorithms in our paper in 30 independent runs on each test problem, which are in the form of box plots [49]. In a notched box plot, the notches represent a robust estimate of the uncertainty about the medians for box-to-box comparison. Symbol “+” denotes outlier (Figure 4).

The 30-variable ZDT1 problem has a convex Pareto-optimal front, while ZDT2 has a nonconvex Pareto-optimal front, and ZDT3 provides difficulties by its discontinuities. Many MOEAs have achieved very good results on these problems in both goals of multiobjective optimization (convergence to the true Pareto front and uniform spread of solutions along the front). The results of the problems ZDT1, ZDT2, and ZDT3 (Figures 3 and 4) show that MMEDA achieves good results, which are comparable to the results of RM-MEDA, GDE3, and PCX-NSGA-II; however, MMEDA is not the best in all metrics of the four algorithms. On the first three test problems we cannot see a meaningful difference in performance of the four algorithms. If it is exigent to find which is best, we can see that RM-MEDA is slightly better than the other three algorithms in terms of spacing, while it performs a little poor in convergence metric. Besides, MMEDA and GDE3 are a little better in terms of convergence metric, while they achieve a little worse in spacing metric of these three problems.

**Figure 5:**
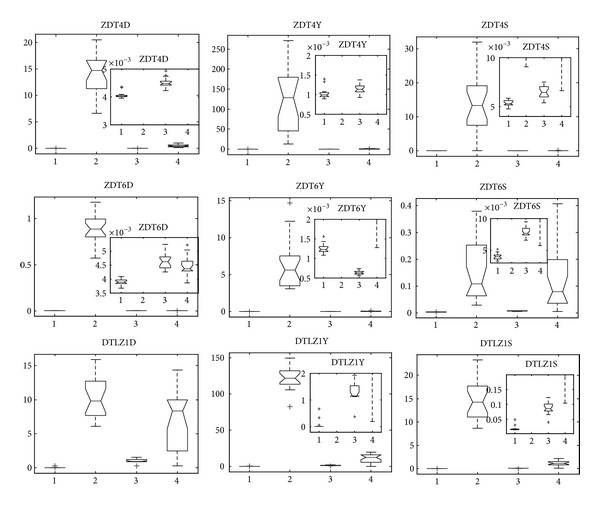
Statistical values of inverted generation distance (“D” for short), convergence (“Y” for short), and spacing (“S” for short) for ZDT4, ZDT6, and DTLZ1 obtained by MMEDA, RM-MEDA, GDE3, and PCX-NSGA-II, respectively.

ZDT4 is a hard optimization problem with many (21^9^) local Pareto fronts that tend to mislead the optimization algorithm. In Figure 5, we can see that RM-MEDA has difficulty in locating the global true Pareto-optimal front. Besides, MMEDA, GDE3, and PCX-NSGA-II produce a very similar convergence and diversity measure. Furthermore, it seems that MMEDA demonstrates the best in terms of convergence and diversity by box plots in small scale.

With respect to the 10-variable test problem ZDT6, there are two major difficulties. The first one is thin density of solutions towards the Pareto front and the second one is nonuniform spread of solutions along the front. The Pareto-optimal solutions of DTLZ1 with (11^5^ – 1) local Pareto fronts lie on a three-dimensional plane satisfying *f*_1_ + *f*_2_ + *f*_3_ = 0.5 (Figure 3). In Figure 5, we can obtain that MMEDA and GDE3 achieve good convergence and diversity measures of ZDT6, followed by PCX-NSGA-II and RM-MEDA, while, for DTLZ1, it seems that MMEDA gives both the best convergence and diversity measure in the four algorithms.

On the whole, we can obtain that MMEDA seems to be the best algorithm on these three problems. However, if we do not employ local searcher, EDAs based on probability global statistical information may perform weakly in approximating the Pareto-optimal fronts of the three problems.

**Figure 6:**
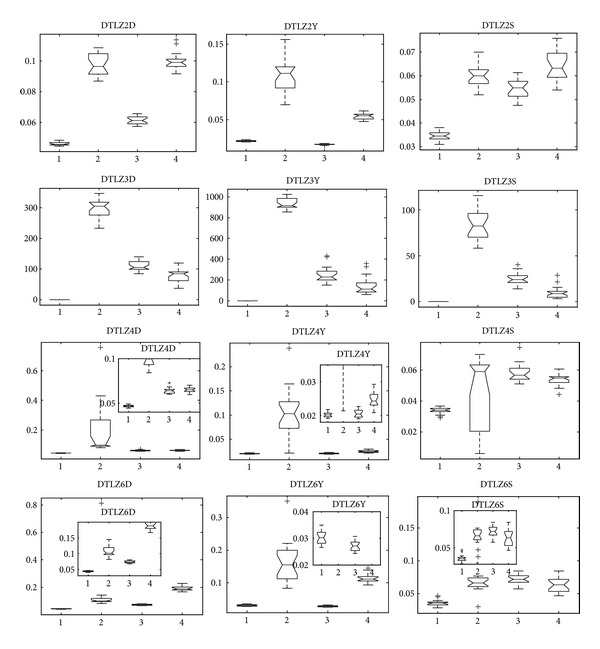
Statistical values of inverted generation distance (“D” in DTLZXD for short), convergence (“Y” in DTLZXY for short), and spacing (“S” in DTLZXS for short) for DTLZ2, DTLZ3, DTLZ4, and DTLZ6 obtained by MMEDA, RM-MEDA, GDE3, and PCX-NSGA-II, respectively.

Problems of DTLZ2, DTLZ3, and DTLZ4 are three-objective test instances with spherical Pareto-optimal front (see Figure 3). Note that DTLZ3 has lots of local Pareto fronts, DTLZ4 emphasizes nonuniformity, and DTLZ6 has 2^19^ disconnected Pareto-optimal regions in the search space.  Figure 6 shows the performance metrics of the four algorithms on the four problems, respectively. We can find that MMEDA is the best in terms of both convergence and diversity on DTLZ3 and DTLZ4. Furthermore, for DTLZ3, MMEDA performs much better than the other there algorithms even though DTLZ3 has (3^10^ – 1) local Pareto-optimal fronts. Gong et al. [9], Deb et al. [37], and Khare et al. [46] claimed that, for DTLZ3, NSGA-II, SPEA2, and PESA-II could not quite converge on to the true Pareto-optimal fronts in 500 generations (50 000 function evaluations). In our paper, we have found that GDE3, PCX-NSGA-II, and RM-MEDA also did badly in solving DTLZ3.

For convergence metric, it seems that GDE3 achieves the best measure in the four algorithms on DTLZ2 and DTLZ6. Besides, for diversity metric, MMEDA is the best on the four problems, which is the strongpoint of *ε*-dominance. Since the *ε*-dominance does not allow two solutions with a difference of *ε*_*i*_ in the *i*th objective to be mutually nondominated to each other, it will be usually not possible to obtain the extreme corners of the Pareto-optimal front. Although there is such shortcoming of *ε*-dominance, we could still get better spacing measurement than that of RM-MEDA, GDE3, and PCX-NSGA-II, which are based on crowding distance proposed by Deb et al. [6].

Overall considering the ten famous two and three objective problems without variable linkages, we can conclude that MMEDA produces a good convergence and diversity metrics with the exception of ZDT1, ZDT2, and ZDT3. GDE3 obtains comparative results for most of problems except DTLZ1 and DTLZ3. Besides, PCX-NSGA-II and RM-MEDA are not better than MMEDA and GDE3 on most of the ten problems. However, the mechanism of RM-MEDA is based on global statistical information, and strongpoints of RM-MEDA are discovering linkages of variables and holding the spread of the final solutions. Besides, there are no variable linkages in the former ten problems. Therefore, it is not fair for RM-MEDA. In [14], it has been validated that RM-MEDA performs better than GDE3 and PCX-NSGA-II on some multiobjective problems with variable linkages. In the next subsection, we will give experimental results of our algorithms compared with the other three algorithms on twelve multiobjective problems with variable linkages.

### 6.6. Experimental Results of Multiobjective Problems with Variable Linkages

In this subsection, experimental results of twelve multiobjective problems with variable linkage are presented. The representation of experimental results is similar to that of Section 6.5; that is, 30 independent runs are performed on each test problem. The statistical results of our selected three metrics are shown by box plots too.

**Figure 7:**
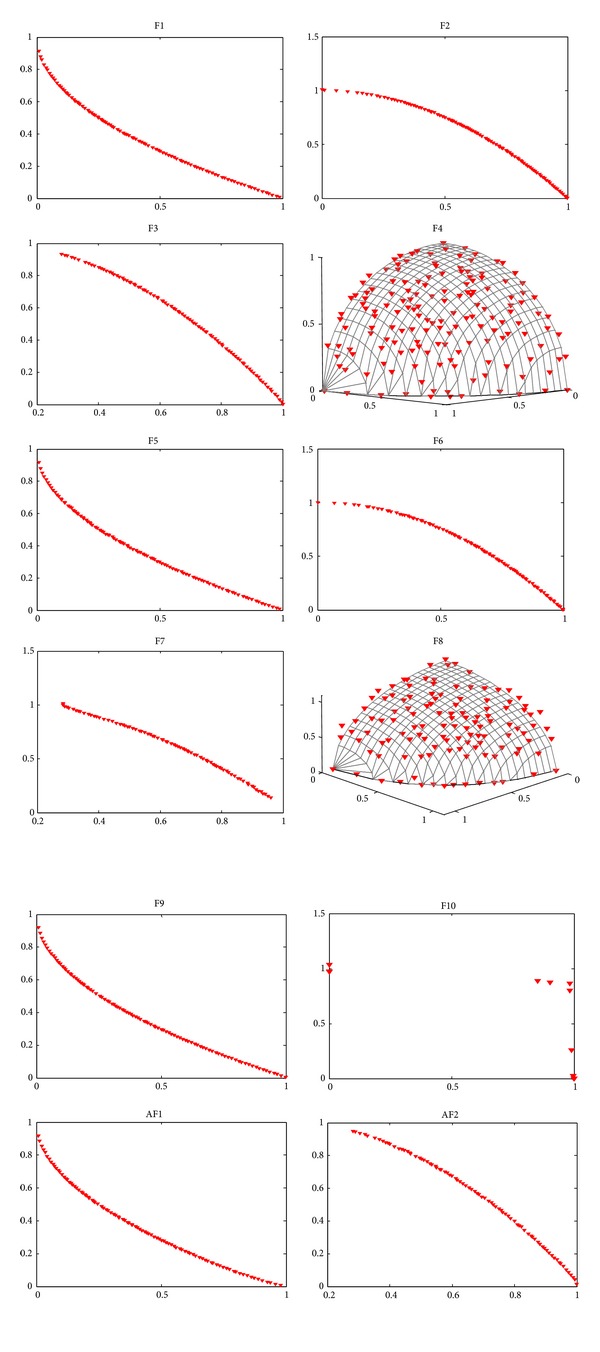
Pareto fronts obtained by MMEDA on F1~F10 and AF1, AF2 in our paper, respectively.

Figure 7 shows the Pareto fronts obtained by our algorithm on F1~F10 and AF1, AF2 in a random single run. We can see that MMEDA obtains good results of F1, F2, F3, F4, F5, F6, F9, AF1, and AF2. However, MMEDA performs a little poor on F7, F8, and F10, especially on F10, of which MMEDA cannot converge to the true Pareto-optimal fronts. In [14], RM-MEDA is also trapped in local Pareto front on F10. Furthermore, for GDE3 and PCX-NSGA-II, they converge to a small region of global Pareto fronts of F10, which is called “genetic drift.” Next, we will give experimental comparison with MMEDA, RM-EDA, GDE3, and PCX-NSGA-II by box plots for further research.

**Figure 8:**
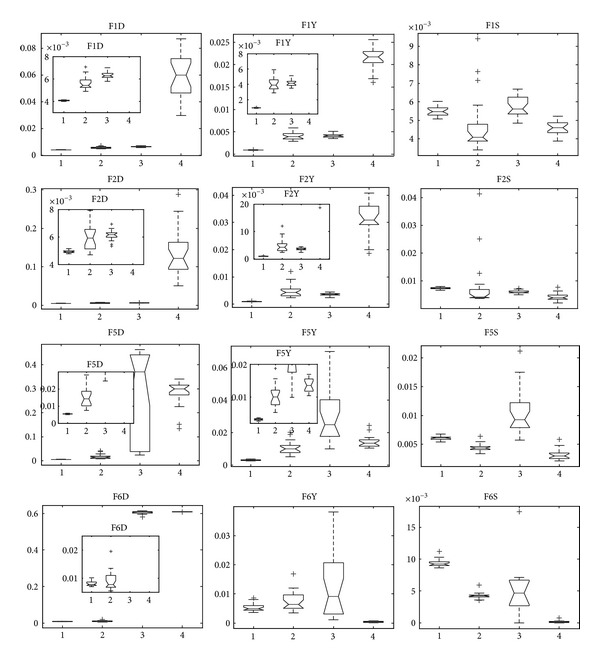
Statistical values of inverted generation distance (“D” in FXD for short), convergence (“Y” in FXY for short), and spacing (“S” in FXS for short) for F1, F5, F2, and F6 obtained by MMEDA, RM-MEDA, GDE3, and PCX-NSGA-II, respectively.

Since F1 and F5 are variants of ZDT1 by introducing linear and nonlinear variable linkages into them, respectively, we put the statistical results of them in the same figure (Figure 8) for comparison, which are similar to F2 and F6. Note that the metric inverted generation distance should be first choice for comparison because inverted generation distance can measure both convergence and spread. If we ignore inverted generation distance, we cannot get rational and comprehensive comparison. Taking F6, for example, if we only concentrate on the metric convergence and spacing, it seems that PCX-NSGA-II is the best choice for this problem. Nevertheless, analyzing F6D in Figure 8, the result of F6 is in a small region of whole Pareto- optimal front, which is called “genetic drift” by Goldberg and Segrest [50] and Fonseca and Fleming [51]. Zhang et al. got the same results of PCX-NSGA-II on F6 [14]. For this end, inverted generation distance is foundational one among the three metrics.

In terms of convergence metric of F1 and F2, MMEDA obtains best performance, while it performs a little poor in spacing metric. Furthermore, MMEDA and RM-MEDA perform better than GDE3 and PCX-NSGA-II on the four problems in terms of spread and convergence. Although PCX-NSGA-II seems better in spacing metric on F5 and F6, it experiences difficulty in spread.

**Figure 9:**
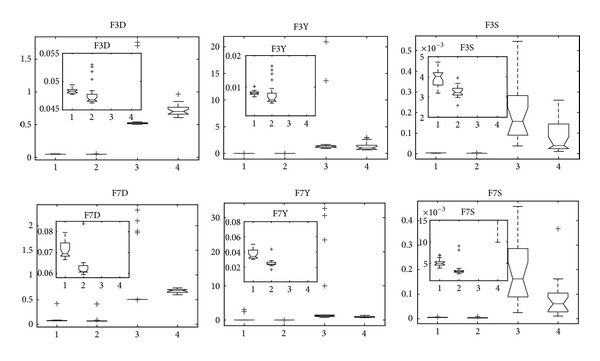
Statistical values of inverted generation distance (“D” in FXD for short), convergence (“Y” in FXY for short), and spacing (“S” in FXS for short) for F3 and F7 obtained by MMEDA, RM-MEDA, GDE3, and PCX-NSGA-II, respectively.

With respect to F3 and F7, since they are variants of ZDT6 by introducing linear and nonlinear variable linkages into them, respectively, there are three major difficulties of them. The first two difficulties are the same as ZDT6 as described above, while the third one is linkage relations between variable mappings. Therefore, F3 and F7 have more difficulty than F1, F2, F5, and F6.  Figure 9 shows the performance measures on F3 and F7. We can obtain that RM-MEDA and MMEDA achieve better performance than GDE3 and PCX-NSGA-II in terms of spread, convergence, and uniformity. This might be the reason why GDE3 and PCX-NSGA-II have no efficient mechanism for discovering variable relation, yet RM-MEDA and MMEDA hold the property by building the probability distribution model based on manifold distribution of Pareto set in decision space, which have been described in detail in Section 3. Finally, it seems that RM-MEDA is slightly better than MMEDA on the two problems.

**Figure 10:**
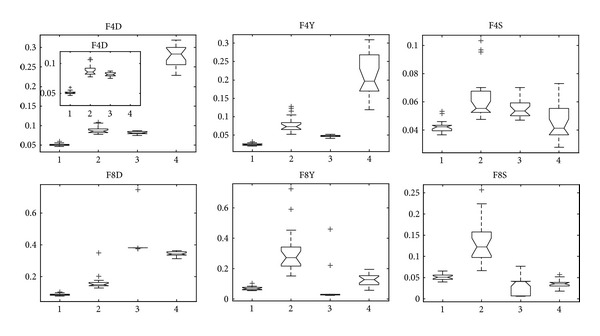
Statistical values of inverted generation distance (“D” in FXD for short), convergence (“Y” in FXY for short), and spacing (“S” in FXS for short) for F4 and F8 obtained by MMEDA, RM-MEDA, GDE3, and PCX-NSGA-II, respectively.

**Figure 11:**
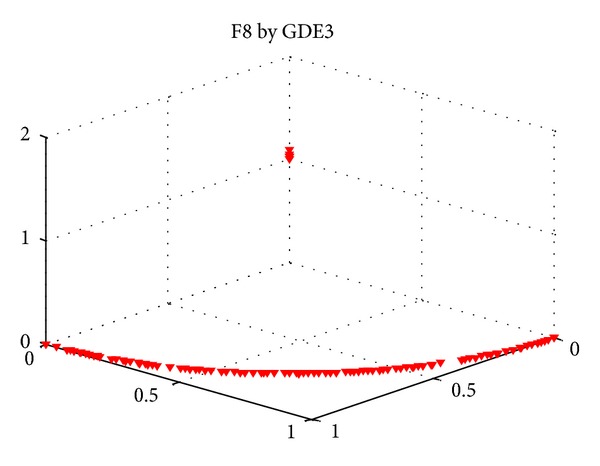
Pareto fronts obtained by GDE3 on F8 in a single run.

F4 and F8 are variants of DTLZ2, and Figure 10 is the statistical results of them. We can obtain that MMEDA is slightly better than other three algorithms in our paper. Besides, GDE3 shows comparative results on F4, followed by RM-MEDA. In [14], Zhang et al. admitted that GDE3 slightly outperforms better than RM-MEDA on some problems and pointed out that the reason might be that RM-MEDA does not directly use the local information of previous solutions in generating new solutions. Here, we give one possible answer, MMEDA, the hybridization of RM-MEDA and an efficient local searcher. For F8, GDE3 and PCX-NSGA-II both converge to a curve and several points, which is “genetic drift” in multiobjective optimization.  Figure 11 is the result of a single run of GDE3, and PCX-NSGA-II has similar results with GDE3. In multiobjective optimization, genetic drift means that finite population tends to converge to small regions of efficient set.

**Figure 12:**
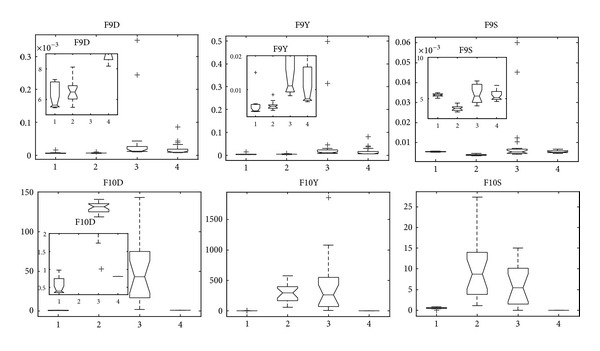
Statistical values of inverted generation distance (“D” in FXD for short), convergence (“Y” in FXY for short), and spacing (“S” in FXS for short) for F9 and F10 obtained by MMEDA, RM-MEDA, GDE3, and PCX-NSGA-II, respectively.

F9 is a variant of multimodal with Griewank function, while F10 is a variant of multimodal with Rastrigin function, and all of them are introduced into nonlinear mapping on variables.  Figure 12 is the statistical results of them by the four algorithms in our study. We can obtain that MMEDA and RM-MEDA get very similar performance of F9, and they get better measurement than GDE3 and PCX-NSGA-II. However, with respect to F10, it seems that MMEDA and PCX-NSGA-II are the best two algorithms in terms of the three metrics. However, it is not true to them. In Figure 7 (F10), we can see that all the solutions obtained by MMEDA converge to a small region near the global Pareto-optimal front, which is similar to PCX-NSGA-II [14]. They are trapped in “genetic drift.” Besides, the final Pareto fronts obtained by RM-MEDA and GDE3 on F10 are far from the Pareto-optimal front, which can be validated by inverted generation distance (F10D in Figure 12) and convergence metric (F10Y in Figure 12). They are stagnated at local Pareto fronts. In a word, we have to admit that all the four algorithms cannot solve the problem efficiently.

**Figure 13:**
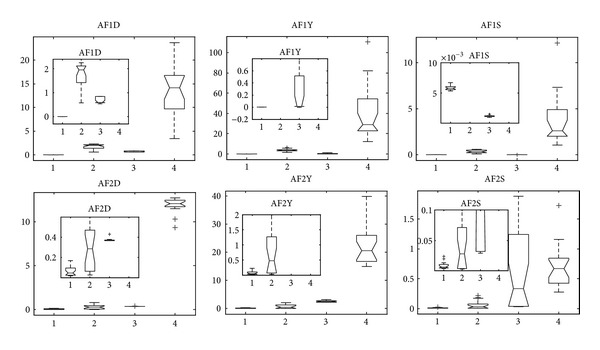
Statistical values of inverted generation distance (“D” in AFXD for short), convergence (“Y” in AFXY for short), and spacing (“S” in AFXS for short) for AF1 and AF2 obtained by MMEDA, RM-MEDA, GDE3, and PCX-NSGA-II, respectively.

By comparison, we can see that MMEDA is best in convergence and spread on AF1 and AF2, followed by RM-MEDA and GDE3. For PCX-NSGA-II (Figure 13), it cannot converge to the true Pareto-optimal front of the two problems. From these two problems, we can see that MMEDA, which hybrids with ITLS, is a competitive algorithm for some problems.

However, overall considering the experimental results of the twelve problems with variable linkages, RM-MEDA and MMEDA are two more efficient and effective algorithms than traditional EAs for most of the problems. For EDAs, this new class of algorithms generalizes genetic algorithms by replacing the crossover and mutation operators by learning and sampling the probability distribution of the promising individuals of population, and the relationships between the variables involved in the problem domain are explicitly and effectively captured and exploited. Besides, the regularity that the distribution of Pareto set in the decision space is a piecewise continuous (*m* − 1)-dimensional manifold is hybrid into MMEDA and RM-MEDA. Therefore, we can conclude that MMEDA and RM-MEDA should be better GDE3 and PCX-NSGA-II. Besides, from two problems (F3, F7) we can see that RM-MEDA is better than MMEDA in all metrics. However, in terms of convergence, MMEDA is slightly better than RM-MEDA on nine problems (F1, F2, F4, F5, F6, F8, F10, AF1, and AF2) and the effectiveness of our ITLS seems to play a very significant role for the above nine problems. But, for seven problems (F1~F3, F5~F7, and F9), RM-MEDA outperforms MMEDA in terms of spacing metric.

As we know, convergence to the true Pareto-optimal front and maintenance of a well-distributed set of nondominated solutions are two challenges for multiobjective optimization community. However, if solutions obtained by an algorithm cannot converge to the true Pareto-optimal fronts, they are stagnated in local optimal fronts. It is useless to consider uniformity. Besides, if solutions obtained by an EMOA only converge to a small region of the whole Pareto-optimal front, they have trouble in “genetic drift.” We cannot make a comprehensive view of the EMOA if we only take convergence and spacing metrics into account. Since the inverted generation can measure both convergence and spread of solutions obtained by an EMOA, it is used as remedy for the shortcomings of generation distance and spacing metrics. Next, we judge the statistical results by combined metrics.

Finally, with respect to the twenty-two multiobjective problems with and without variable linkages, we can conclude the following.

1. In terms of convergence and spread, MMEDA achieves the best measurement among the four algorithms on ZDT4, DTLZ1, DTLZ3, DTLZ4, F1, F2, F4, F5, F8, F9, AF1, and AF2 out of the twenty-two problems while RM-MEDA performs best on F3, F6, and F7. GDE3 does best on ZDT1, ZDT3, ZDT6, DTLZ2, and DTLZ6. PCX-NSGA-II seems to obtain the best result of ZDT2.
2. In terms of spread and spacing, MMEDA performs the best performance on ZDT4, ZDT6, DTLZ1, DTLZ2, DTLZ3, DTLZ4, DTLZ6, F4, F8, AF1, and AF2, while RM-MEDA is the best on ZDT1, ZDT2, ZDT3, F1, F3, F5, F6, F7, and F9. GDE3 achieves the best on F2.

Overall considering the three metrics, we can get that MMEDA turns out to be the best compromise among the four MOEAs considered in this study. However, MMEDA is possibly trapped in “genetic drift,” and we can get it from the results of F10 in Figure 3. Therefore, much work is needed to balance the exploration ability of global model and the exploitation ability of local searcher, or more efficient EDAs based on other machine learning techniques should be proposed.

In one word, depending on these empirical results and detailed analysis, we can draw that MMEDA is an efficient algorithm in solving multiobjective optimization problems with and without variable linkages.

## 7. Concluding Remarks

We have proposed a novel multiobjective algorithm based on the manifold distribution of Pareto set in the decision space, an efficient local searcher (ITLS), and *ε*-dominance. Besides, two more difficult multiobjective problems by introducing nonlinear mapping into variable linkages are proposed. Following Deb et al. and Zhang et al.'s recent review of current evolutionary multiobjective optimization fields [13, 14] multiobjective problems with variable linkages have more difficulty for most current state-of-the-art MOEAs. Consequently, in order to test our proposed algorithm, MMEDA, twenty-two multiobjective problems with and without variable linkages are employed, and RM-MEDA, GDE3, and PCX-NSGA-II are used in comparison. The results show that our algorithms get competitive results in terms of convergence and diversity metrics.

However, there is much work to do in multiobjective optimization community. An issue that should be addressed in the future research is *ε*-dominance. In our study, it seems that spacing metric of some two-objective problems (such as ZDT1 and ZDT3) is not better than PCX-NSGA-II and GDE3, which are based on crowding distance in SBX-NSGA-II [6], and Deb et al. [31] have validated by experiments that *ε*-MOEAs get better spacing metric than NSGA-II based on crowding distance. There are two major reasons of this. The first is that we introduce a local searcher (ITLS) and the number of iterations will be less than the other three algorithms in our study under the same limit of total evaluations. The second is that some extreme points of the Pareto front, as well as points located in segments of the Pareto front that are almost horizontal or vertical, are lost. This is the curse of *ε*-dominance. Some scholars have proposed Pareto adaptive *ε*-dominance [48], which is an advanced version of *ε*-dominance. But the loss of extreme solutions still exists. So much work is still needed to propose new version of dominance.

Another important topic is “genetic drift,” which has been studied by some researchers, such as Goldberg and Segrest [50], Fonseca and Fleming [51], and Srinivas and Deb [52]. At first, fitness sharing techniques are proposed to prevent genetic drift in multimodal function optimization and multiobjective optimization [52]. Later on, fitness assignment and crowding distance are proposed by Deb et al. [6] and Zitzler et al. [7] for diversity maintenance. Nowadays, some state-of-the-art MOEAs have still trouble in “genetic drift” on some multiobjective problems with variable linkages [13, 14]. So how we can design new efficient and effective scheme to maintain diversity is an important issue of modern EMO community.

How to advance the local search ability of modern MOEAs is another worthwhile work. Gong et al. [9], Deb et al. [37], and Khare et al. [46] validated that, for DTLZ3, NSGA-II, SPEA2, and PESA-II, which are representative of state-of-the-art MOEAs in multiobjective optimization community, could not quite converge on to the true Pareto-optimal fronts in 500 generations (50 000 function evaluations). We have found that GDE3, PCX-NSGA-II, and RM-MEDA also did badly in solving DTLZ3. DTLZ3 may be only one case of problems with many local Pareto fronts, and, possibly, there are many complicated problems like DTLZ3. Therefore, it requires designing more efficient and effective algorithms. Memetic algorithm may be one potential answer to the problem, and several multiobjective memetic algorithms have been proposed by some scholars [27, 29, 53], which have shown comparative performance than traditional EMOAs. A more efficient and effective hybrid algorithm should be addressed in the future research.

## Figures and Tables

**Algorithm 1:**
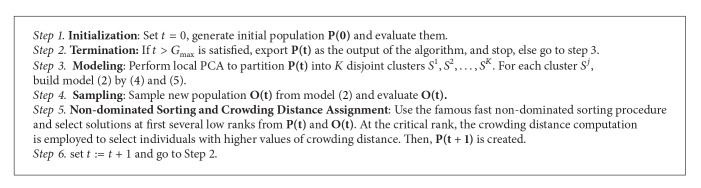
The main loop of RM-MEDA.

**Algorithm 2:**
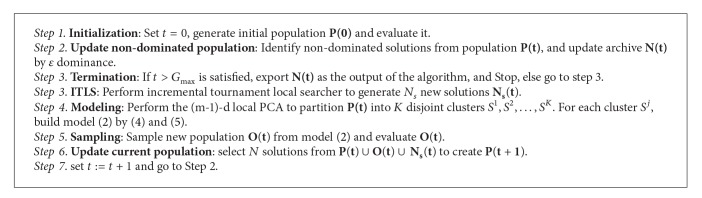
The details of MMEDA.

**Table 1:** Test instances.

Instance	Variable	Objectives	Number of evaluations
ZDT1	[0,1]^*n*^ *n* = 30	$\begin{array}{c} f_{1}(x)=x_{1}\\ f_{2}\left(x\right)=g\left(x\right)\left[1-\sqrt{f_{1}\left(x\right)/g\left(x\right)}\right]\\ g\left(x\right)=1+9\left(\sum_{i=2}^{n}x_{i}\right)/\left(n-1\right) \end{array}$	50 000

ZDT2	[0,1]^*n*^ *n* = 30	*f*_1_(**x**) = *x*_1_ *f*_2_(**x**) = *g*(**x**)[1 − (*f*_1_(**x**)/*g*(**x**))^2^] *g*(**x**) = 1 + 9(∑_*i*=2_^*n*^ *x*_*i*_)/(*n* − 1)	50 000

ZDT3	[0,1]^*n*^ *n* = 30	*f*_1_(**x**) = *x*_1_ $f_{2}\left(x\right)=g\left(x\right)\left[1-\sqrt{f_{1}\left(x\right)/g\left(x\right)}-f_{1}(x)/g\left(x\right)\sin\left(10\pi x_{1}\right)\right]$ *g*(**x**) = 1 + 9(∑_*i*=2_^*n*^ *x*_*i*_)/(*n* − 1)	50 000

ZDT4	*x*_1∈[0,1]_ *x*_*i*∈[−5,5]_ *i* = 2,…, 10	*f*_1_(**x**) = *x*_1_ $f_{2}\left(x\right)=g\left(x\right)\left[1-\sqrt{f_{1}\left(x\right)/g\left(x\right)}\right]$ *g*(**x**) = 1 + 10(*n* − 1) + ∑_*i*=2_^*n*^[*x*_*i*_^2^ − 10cos⁡(4*πx*_*i*_)]	50 000

ZDT6	[0,1]^*n*^ *n* = 10	*f*_1_(**x**) = 1 − exp⁡(−4*x*_1_)sin⁡^6^⁡(6*πx*_1_) *f*_2_(**x**) = *g*(**x**)[1 − (*f*_1_(**x**)/*g*(**x**))^2^] *g*(**x**) = 1 + 9[(∑_*i*=2_^*n*^ *x*_*i*_)/(9)]^0.25^	50 000

DTLZ1	[0,1]^*n*^ *n* = 7 **x**_*M*_ = [0,1]^5^	$\begin{array}{c} f_{1}\left(x\right)=0.5x_{1}x_{2}\left(1+g\left(x\right)\right)\\ f_{2}\left(x\right)=0.5x_{1}\left(1-x_{2}\right)\left(1+g\left(x\right)\right)\\ f_{3}\left(x\right)=0.5\left(1-x_{1}\right)\left(1+g\left(x\right)\right)\\ g\left(x\right)=100\left[\left|x_{M}\right|+\sum_{x_{i}\in X_{M}}^{}\left({\left(x_{i}-0.5\right)}^{2}-\cos\left(20\pi \left(x_{i}-0.5\right)\right)\right)\right] \end{array}$	60 000

DTLZ2	[0,1]^*n*^ *n* = 12	$\begin{array}{c} f_{1}(x)=\cos\left(0.5\pi x_{1}\right)\cos\left(0.5\pi x_{2})(1+g\right)\\ f_{2}(x)=\cos\left(0.5\pi x_{1}\right)\sin\left(0.5\pi x_{2})(1+g\right)\\ f_{3}(x)=\sin\left(0.5\pi x_{1})(1+g\right)\\ g\left(x\right)={\sum_{i=3}^{n}\left(x_{i}-0.5\right)}^{2} \end{array}$	50 000

DTLZ3	[0,1]^*n*^ *n* = 12	$\begin{array}{c} f_{1}(x)=\cos\left(0.5\pi x_{1}\right)\cos\left(0.5\pi x_{2})(1+g\right)\\ f_{2}(x)=\cos\left(0.5\pi x_{1}\right)\sin\left(0.5\pi x_{2})(1+g\right)\\ f_{3}(x)=\sin\left(0.5\pi x_{1})(1+g\right)\\ g\left(x\right)=100\left[\left|x_{M}\right|+\sum_{i=3}^{n}\left({\left(x_{i}-0.5\right)}^{2}-\cos\left(20\pi \left(x_{i}-0.5\right)\right)\right)\right] \end{array}$	50 000

DTLZ4	[0,1]^*n*^ *n* = 12	$\begin{array}{c} f_{1}(x)=\cos\left(0.5\pi {x_{1}}^{\alpha }\right)\cos(0.5\pi {x_{2}}^{\alpha })(1+g)\\ f_{2}(x)=\cos\left(0.5\pi {x_{1}}^{\alpha }\right)\sin\left(0.5\pi {x_{2}}^{\alpha })(1+g\right)\\ f_{3}(x)=\sin\left(0.5\pi {x_{1}}^{\alpha })(1+g\right)\\ g\left(x\right)={\sum_{i=3}^{n}\left(x_{i}-0.5\right)}^{2}\begin{array}{cc} & \alpha =100 \end{array} \end{array}$	50 000

DTLZ6	[0,1]^*n*^ *n* = 22 **x**_*M*_ = [0,1]^20^	$\begin{array}{c} f_{1}(x)=x_{1}\begin{array}{cc} & f_{2}(x)=x_{2} \end{array}f_{3}(x)=\left(1+g\left(x_{M}\right)\right)h\\ g\left(x_{M}\right)=1+0.45\sum x_{i}\begin{array}{cc} & x_{i}\in x_{M} \end{array}\\ h=3-f_{1}/\left(1+g\right)\left(1+\sin\left(3\pi f_{1}\right)\right)-f_{2}/\left(1+g\right)\left(1+\sin\left(3\pi f_{2}\right)\right) \end{array}$	50 000

F1	[0,1]^*n*^ *n* = 30	$\begin{array}{c} f_{1}(x)=x_{1}\begin{array}{cc} & f_{2}\left(x\right)=g\left(x\right)\left[1-\sqrt{f_{1}\left(x\right)/g\left(x\right)}\right] \end{array}\\ g\left(x\right)=1+9\left(\sum_{i=2}^{n}{\left(x_{i}-x_{1}\right)}^{2}\right)/\left(n-1\right) \end{array}$	15 000

F2	[0,1]^*n*^ *n* = 30	$\begin{array}{c} f_{1}(x)=x_{1}\begin{array}{cc} & f_{2}\left(x\right)=g\left(x\right)\left[1-{\left(f_{1}\left(x\right)/g\left(x\right)\right)}^{2}\right] \end{array}\\ g\left(x\right)=1+9\left(\sum_{i=2}^{n}{\left(x_{i}-x_{1}\right)}^{2}\right)/\left(n-1\right) \end{array}$	15 000

F3	[0,1]^*n*^ *n* = 30	*f*_1_(**x**) = 1 − exp⁡(−4*x*_1_)sin⁡^6^⁡(6*πx*_1_) *f*_2_(**x**) = *g*(**x**)[1 − (*f*_1_(**x**)/*g*(**x**))^2^] *g*(**x**) = 1 + 9[(∑_*i*=2_^*n*^(*x*_*i*_−*x*_1_)^2^)/9]^0.25^	100 000

F4	[0,1]^*n*^ *n* = 30	$\begin{array}{c} f_{1}(x)=\cos\left(0.5\pi x_{1}\right)\cos\left(0.5\pi x_{2})(1+g\right)\\ f_{2}(x)=\cos\left(0.5\pi x_{1}\right)\sin\left(0.5\pi x_{2})(1+g\right)\\ f_{3}(x)=\sin\left(0.5\pi x_{1})(1+g\right)\\ g\left(x\right)={\sum_{i=3}^{n}\left(x_{i}-x_{1}\right)}^{2} \end{array}$	35 000

F5	[0,1]^*n*^ *n* = 30	$\begin{array}{c} f_{1}(x)=x_{1}\begin{array}{cc} & f_{2}\left(x\right)=g\left(x\right)\left[1-\sqrt{f_{1}\left(x\right)/g\left(x\right)}\right] \end{array}\\ g\left(x\right)=1+9\left(\sum_{i=2}^{n}{\left({x_{i}}^{2}-x_{1}\right)}^{2}\right)/\left(n-1\right) \end{array}$	15 000

F6	[0,1]^*n*^ *n* = 30	$\begin{array}{c} f_{1}(x)=x_{1}\begin{array}{cc} & f_{2}\left(x\right)=g\left(x\right)\left[1-{\left(f_{1}\left(x\right)/g\left(x\right)\right)}^{2}\right] \end{array}\\ g\left(x\right)=1+9\left(\sum_{i=2}^{n}{\left({x_{i}}^{2}-x_{1}\right)}^{2}\right)/\left(n-1\right) \end{array}$	15 000

F7	[0,1]^*n*^ *n* = 30	$\begin{array}{c} f_{1}\left(x\right)=1-\exp\left(-4x_{1}\right)\sin^{6}\left(6\pi x_{1}\right)\\ f_{2}\left(x\right)=g\left(x\right)\left[1-{\left(f_{1}\left(x\right)/g\left(x\right)\right)}^{2}\right]\\ g\left(x\right)=1+9{\left[\left(\sum_{i=2}^{n}{\left({x_{i}}^{2}-x_{1}\right)}^{2}\right)/9\right]}^{0.25} \end{array}$	100 000

F8	[0,1]^*n*^ *n* = 30	$\begin{array}{c} f_{1}(x)=\cos\left(0.5\pi x_{1}\right)\cos(0.5\pi x_{2})(1+g)\\ f_{2}(x)=\cos\left(0.5\pi x_{1}\right)\sin\left(0.5\pi x_{2})(1+g\right)\\ f_{3}(x)=\sin\left(0.5\pi x_{1})(1+g\right)\\ g\left(x\right)={\sum_{i=3}^{n}\left({x_{i}}^{2}-x_{1}\right)}^{2} \end{array}$	35 000

F9	[0,1] × [0,10]^*n*−1^ *n* = 30	$\begin{array}{c} f_{1}(x)=x_{1}\begin{array}{cc} & f_{2}\left(x\right)=g\left(x\right)\left[1-\sqrt{f_{1}\left(x\right)/g\left(x\right)}\right] \end{array}\\ g(x)=0.00025\sum_{i=2}^{n}{\left(x_{i}^{2}-x_{1}\right)}^{2}-\prod_{i=2}^{n}\cos\left(\left({x^{2}}_{i}\right)/\sqrt{i-1}\right)+2 \end{array}$	100 000

F10	[0,1] × [0,10]^*n*−1^ *n* = 30	$\begin{array}{c} f_{1}(x)=x_{1}\begin{array}{cc} & f_{2}\left(x\right)=g\left(x\right)\left[1-\sqrt{f_{1}\left(x\right)/g\left(x\right)}\right] \end{array}\\ g\left(x\right)=1+10(n-1)+\sum_{i=2}^{n}\left[{\left({x_{i}}^{2}-x_{1}\right)}^{2}-10\cos\left(2\pi \left({x_{i}}^{2}-x_{1}\right)\right)\right] \end{array}$	100 000

AF1	[0,1]^*n*^ *n* = 30	$\begin{array}{c} f_{1}(x)=x_{1}\begin{array}{cc} & f_{2}\left(x\right)=g\left(x\right)\left[1-\sqrt{f_{1}\left(x\right)/g\left(x\right)}\right] \end{array}\\ g\left(x\right)=1+9\left(\sum_{i=2}^{n}{\left(100\sin\left(\pi x_{i}\right)-x_{1}\right)}^{2}\right)/\left(n-1\right) \end{array}$	30 000

AF2	[0,1]^*n*^ *n* = 30	$\begin{array}{c} f_{1}\left(x\right)=1-\exp\left(-4x_{1}\right)\sin^{6}\left(6\pi x_{1}\right)\\ f_{2}\left(x\right)=g\left(x\right)\left[1-{\left(f_{1}\left(x\right)/g\left(x\right)\right)}^{2}\right]\\ g\left(x\right)=1+9{\left[\left(\sum_{i=2}^{n}{\left(6\left(\sin\pi x_{i}-x_{1}\right)\right)}^{2}\right)/9\right]}^{0.25} \end{array}$	60 000

**Table 2:** Indexes of the different algorithms.

Index	1	2	3	4
Algorithms	MMEDA	RM-MEDA	GDE3	PCX-NSGA-II

**Table 3:** The *ε* values for different test instances.

ZDT1	[0.0075, 0.0075]	DTLZ2	[0.045, 0.045, 0.03]	F4	[0.045, 0.045, 0.03]	F10	[0.0075, 0.0075]
ZDT2	[0.0075, 0.0075]	DTLZ3	[0.045, 0.045, 0.03]	F5	[0.0075, 0.0075]	AF1	[0.0075, 0.0075]
ZDT3	[0.0025, 0.0035]	DTLZ4	[0.045, 0.045, 0.03]	F6	[0.0075, 0.0075]	AF2	[0.0075, 0.0075]
ZDT4	[0.0075, 0.0075]	DTLZ6	[0.035, 0.035, 0.035]	F7	[0.0075, 0.0075]	AF3	[0.0075, 0.0075]
ZDT6	[0.0075, 0.0075]	F1	[0.0075, 0.0075]	F8	[0.045, 0.045, 0.03]		
DTLZ1	[0.02, 0.02, 0.05]	F2	[0.0075, 0.0075]	F9	[0.0075, 0.0075]		
